# Three‐Dimensional Change of Molar Position During Alignment and Leveling With PASS and MBT: A Randomized Controlled Trial

**DOI:** 10.1155/ijod/6179629

**Published:** 2026-04-16

**Authors:** Hong Su, Kaiyuan Xu, Zimeng Zhuang, Fei Teng, Tingting Feng, Bing Han, Gui Chen, Tianmin Xu

**Affiliations:** ^1^ First Clinical Division, Peking University School and Hospital of Stomatology, Beijing, 100034, China, bjmu.edu.cn; ^2^ National Center for Stomatology, National Clinical Research Center for Oral Diseases, National Engineering Research Center of Oral Biomaterials and Digital Medical Devices, Beijing Key Laboratory of Digital Stomatology, NHC Key Laboratory of Digital Stomatology, NMPA Key Laboratory for Dental Materials, Beijing, 100081, China, njucm.edu.cn; ^3^ Second Clinical Division, Peking University School and Hospital of Stomatology, Beijing, 100034, China, bjmu.edu.cn; ^4^ Arthur A. Dugoni School of Dentistry, University of the Pacific, Stockton, California, USA, pacific.edu; ^5^ Department of Orthodontics, Peking University School and Hospital of Stomatology, Beijing, 100081, China, bjmu.edu.cn; ^6^ Department of Orthodontics, School of Stomatology, Tongji University, Shanghai Engineering Research Center of Tooth Restoration and Regeneration, 399 Middle Yanchang Road, Jing’an District, Shanghai, 200072, China, tongji.edu.cn

**Keywords:** alignment and leveling, MBT, molar anchorage, PASS, RCT

## Abstract

**Backgrounds:**

With the development of straight wire appliance (SWA), especially the rountine use of nickel‐titanium (NiTi) archwires in the initial stage has largely eliminated the need for bending up. However, engaging fine NiTi wire into the molar buccal tube with built‐in forward tipping prescription can induce unintended mesial tipping and mesialization of maxillary molars, leading to early anchorage loss. This single‐center, two‐arm parallel randomized clinical trial aimed to compare the physiologic anchorage spee‐wire system (PASS) with the Mclaughlin‐Bennet‐Trevici straight‐wire system (MBT) in terms of their ability to preserve maxillary anchorage control during the alignment and leveling.

**Methods:**

Sixty‐four patients with Angle Class I or II malocclusion undergoing maxillary first premolar extraction were recruited. Minimal randomization was used to select the sample. Two bracket systems were used—PASS and MBT. Digital dental models were acquired at pre‐treatment(T0), the first 3 months during treatment(T1), and the first 6 months during treatment(T2). Study models of different time point were superimposed to evaluate movements of maxillary first molars, canines, and central incisors.

**Results:**

During the first 6 months of the treatment, maxillary first molars in the PASS group exhibited distal tipping (2.67° ± 4.59°) and distal movement (0.03 ± 1.02 mm). In contrast, the MBT group showed mesial tipping (1.95° ± 3.04°) and mesial movement (0.67 ± 0.79 mm). Additionally, the maxillary canines also exhibited treatment difference. The increase of inter‐canine width was significantly smaller increased in the PASS group (1.31 ± 2.00 mm) compared to the MBT group (2.37 ± 1.72 mm). No other difference was found.

**Conclusion:**

Compared with the MBT system, PASS was better at preserving maxillary anchorage during the alignment and leveling stage. These findings suggest that PASS offers superior physiological anchorage control in the early stages of treatment without the need for auxiliary appliances.

## 1. Introduction

The primary goals of orthodontic treatment are well‐aligned teeth, ideal occlusion, and a harmonious profile. In Caucasian populations, the extraction rate is relatively low due to the well‐developed nose and chin [1, 2]. Because Andrews developed the straight‐wire appliance (SWA) using a dataset based on normal occlusion without extraction [3], many orthodontists considered the SWA to be ideal for non‐extraction cases.

Considering patient profiles and post‐treatment stability, Tweed [4] argued that the extraction treatment is necessary in many cases. In Asian populations, the extraction rate is significantly higher than that in Western countries due to the prevalence of protrusive profiles [5]. To address both protrusion and crowding, orthodontists who treated patients with SWA have employed various auxiliary devices to reinforce anchorage, including headgear, Nance appliance, and temporary anchorage devices (TADs) [6, 7].

Researches has focused on understanding the etiology of anchorage loss to implement effective preventive strategies. Geron [8] characterized the causes of anchorage loss as multifactorial with determining factors including crowding, mechanics, age, extraction site, and overjet. Su [9] noted that pre‐treatment distal tipping of the maxillary molar is common, and anchorage loss can result from mesial tipping and mesialization during alignment and leveling. Xu [10] proposed that the anchorage loss in extraction cases comprises two parts: mechanical force and physiological force. Based on this concept, Xu conceptualized physiological anchorage loss and developed the Physiological Anchorage Spee‐wire System (PASS) to preserve anchorage by managing both mechanical and physiological forces. A randomized clinical trial (RCT) by Chen [11] demonstrated that the PASS group achieved effective anchorage control without additional anchorage devices by accounting for the dentoalveolar compensation of anchor teeth; however, this study merely evaluated the final treatment result. While molar anchorage loss can occur at any stage of orthodontic treatment, and orthodontists often focus primarily on the space closure stage rather than the initial alignment stage [12]. Although small‐diameter nickel‐titanium (NiTi) wire is effective for aligning tooth, it lacks early molar anchorage control due to poor bending performance. Al‐Awadhi EA [13] found that the majority of anchorage loss occurred during this initial stage, even when a Nance appliance was used. Therefore, a critical question remains: is the PASS technique capable of effectively reduce anchorage loss during the alignment and leveling stage?

This study aimed to compare the PASS and MBT straight‐wire systems regarding their capacity for anchorage control during alignment and leveling. The null hypothesis tested was that the PASS and MBT systems exhibit similar anchorage control capacities.

## 2. Materials and Methods

### 2.1. Trial Design and Any Changes After Trial Commencement

This was a prospective randomized clinical controlled trial (RCT) was conducted in adherence to the Consolidated Standards of Reporting Trials statement and guidelines [14]. No modifications to the methodology were required following trial commencement. The methods have been described in detail in our previous publication [11].

### 2.2. Participants and Settings

The protocol for this study (PKUSSIRB‐2013050) was approved by the Biomedical Ethics Committee of the School and Hospital of Stomatology, Peking University (Beijing, China) and was registered with the Chinese Clinical Trial Registry (Chictr.org.cn) under the identifier ChiCTR‐TRC‐13003260 on June 15, 2013.

This study was part of a series, and the sampling procedures and methods have been detailed in our previous randomized controlled trial [11]. The sample consisted of 64 patients who initiated treatment between June 2013 and July 2014 at the orthodontic department of Peking University School and Hospital of Stomatology. Inclusion criteria in this study were as follows: (1) presence of all erupted permanent teeth, (2) Angle’s Class I or II malocclusion requiring moderate medium or maximum anchorage control, (3) extraction of two upper 1st bicuspids or four 1st bicuspids extracted; and (4) good general health with no severe periodontitis. Patients were excluded if they: (1) required molar distalization to gain extra space, (2) had missing or impacted teeth (excluding the third molars), (3) had posterior crossbite, (4) had a history of orthodontic or surgical treatment; or (5) suffered from systemic diseases, congenital craniofacial deformity and syndromes, or severe periodontitis. Written informed consent was obtained from all patients and their parents or legal guardians prior to recruitment.

### 2.3. Interventions

**Figure 1:**
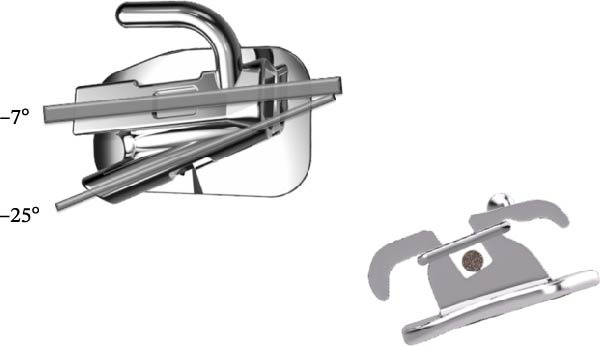
XBT and MLF brackets used in the PASS group. The XBT molar tube consists of two tubes: a distal‐tipping tube that prevents the NiTi wire from tipping the molar forward and a main tube that prevents anchorage loss during space closure. The MLF bracket allows the ligature tie to slide beneath the bracket wings, creating a self‐ligating effect, and enables orthodontists to adjust the amount of friction for each individual tooth as needed.

**Figure 2:**
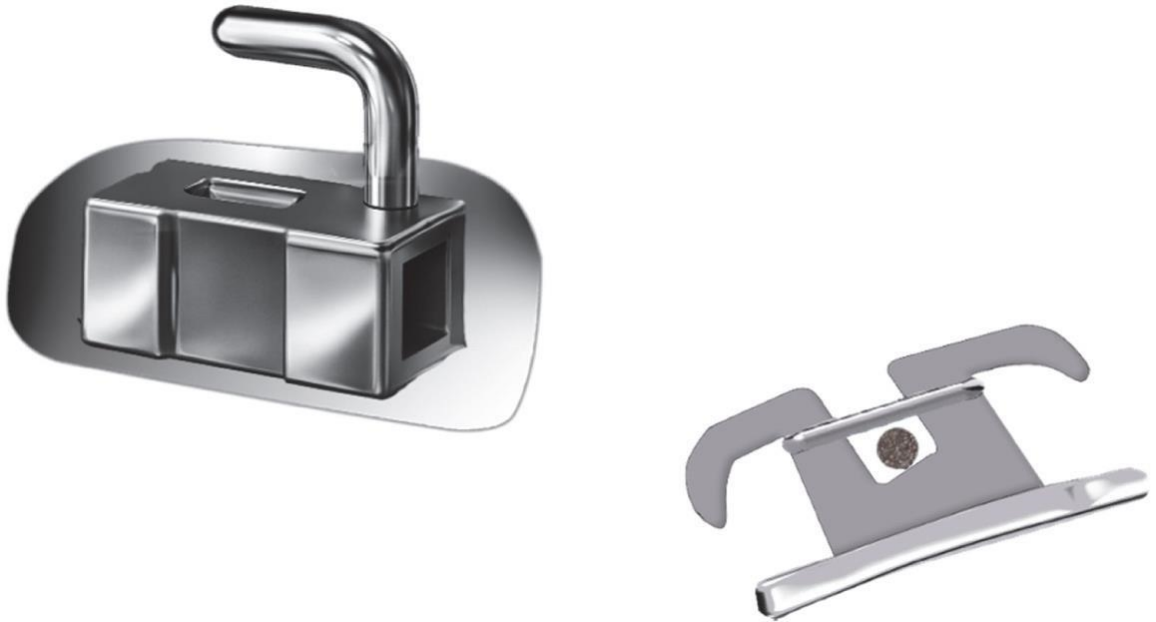
MBT tube that consists of one main tube and bracket used in MBT group.

**Figure 3:**
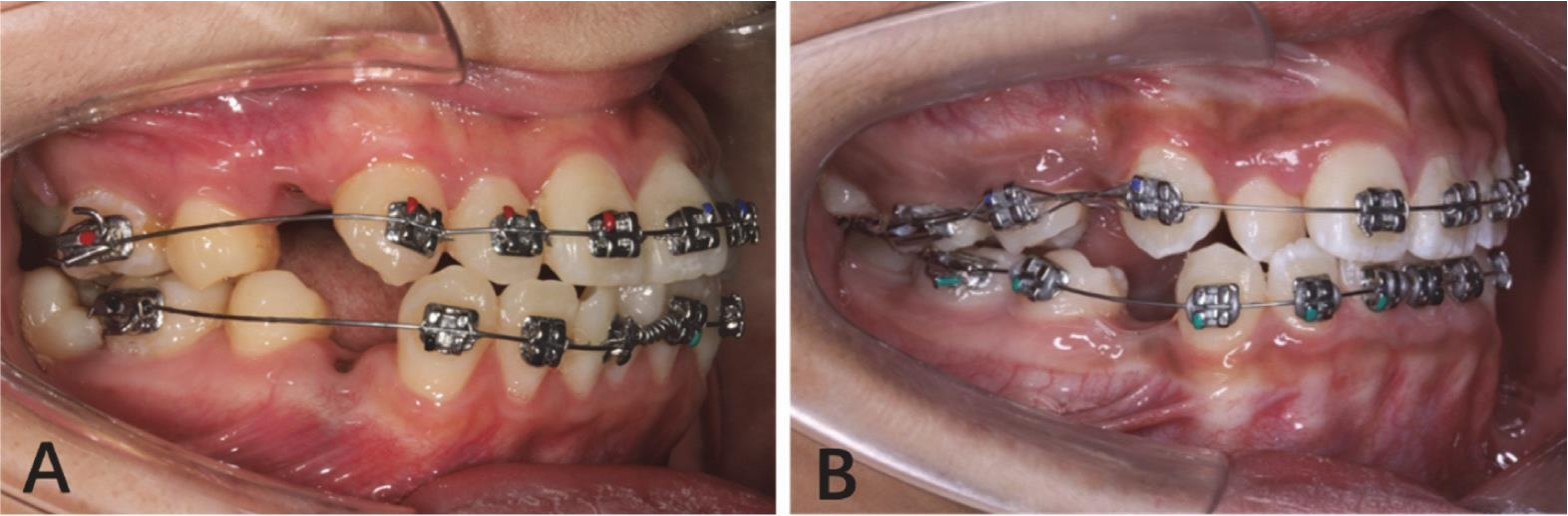
Alignment and leveling procedure using the PASS and MBT appliances. (A) Patients treated with the PASS protocol. (B) Patient treated with the MBT protocol.

The appliance designs and treatment philosophies differ between the PASS (Figure 1) and MBT (Figure 2) [15]. The PASS is a modified straight‐wire system based on the physiological characteristics of the individual patient and classical anchorage control strategies. It aims to reduce maxillary dentoalveolar compensation and shares the similar goal of the Tweed technique regarding the maintenance of the upper curve of Spee is concerned. All archwires and brackets used in this study were manufactured by Shinye (Hangzhou, China). The appliance used in the PASS group is mainly consists primarily of a cross‐buccal tube (XBT) and multi‐level low‐friction (MLF) brackets (Figure 1). The first stage of the treatment in the PASS group focused on the alignment of the anterior teeth. The basic treatment procedure involved bonding canine to canine MLF brackets and placing XBTs on the first molars. Upper and lower 0.014‐inch NiTi archwires were engaged into −25° auxiliary tube on the upper molars and −20° “virtual tube” on lower molars (Figure 3A). Following the anterior alignment, the premolars and second molars were bonded to align the posterior teeth. Subsequently, the main tube was utilized for the remainder of the treatment.

Patients in the MBT group were treated according to standard MBT technique. Preadjusted brackets and buccal tubes with a 0.022 inch slot‐size were bonded [7] (Figure 3B). Upper and lower 0.014 inch NiTi wires were used during the initial leveling and alignment stage. The sequential progression of NiTi archwires was similar in both MBT group and PASS group. Canine lacebacks were used only in MBT group to assist in controlling canine crown during leveling and alignment. Additionally, auxiliary anchorage device were permitted in the MBT group for patients requiring reinforced anchorage control.

### 2.4. Outcomes (Primary and Secondary) and Changes After Trial Commencement

Compared to the mandible, the maxilla is at a higher risk of anchorage loss due to mandible excess growth and lower bone density [16]. Therefore, the three‐dimensional change of the maxillary first molar was designated as the primary outcome measure.

Secondary outcome measures included the 3D changes in the maxillary canines, and maxillary central incisors, specifically evaluating tip, torque, vertical, and antero‐posterior movement (Table 1).

**Table 1:** Definitions of measurements.

Measurements	Definition
Occlusal plane	The best‐fit plane constructed by the bilateral midpoints of the incisal edges of the central incisors and the bilateral mesiobuccal cusp tips of the bilateral first molars on maxillary digital models
6–6 width	Distance between the bilateral mesial buccal cusps of maxillary first molar (projected on the occlusal plane)
6 tip	The tip of maxillary first molar; mesial tipping: “+”
6 tor	The torque of maxillary first molar; buccal crown torque: “+”
6 MCH	The height of the mesial buccal cusp of maxillary first molar; extrusion: “+”
6 DCH	The height of the distal buccal cusp of maxillary first molar; extrusion: “+”
6 AP	The anteroposterior movement of the mesial buccal cusp of maxillary first molar; mesialization: “+”

3–3 width	Distance between the bilateral cusps of maxillary canines (projected on the occlusal plane)
3 tip	The tip of maxillary canine; mesial tipping: “+”
3 tor	The torque of maxillary canine; buccal crown torque: “+”
3 CH	The height of the cusp of maxillary canine; extrusion: “+”
3 AP	The anteroposterior movement of the cusp of maxillary canines; mesialization: “+”

1 tor	The torque of maxillary central incisors；labial crown torque: “+”
1 H	The height of the edge of maxillary central incisor；extrusion: “+”
1 AP	The anteroposterior movement of the edge of maxillary central incisor；mesialization: “+”

All measurements were performed on digital dental models obtained at pre‐treatment(T0), 3 months(T1), and 6 months(T2) using Rapidform 2006 (Inus Technology Inc., Seoul, Korea). Maxillary digital models from the different time points were superimposed according to Chen’s method [17] (Figure 4A). A reference coordinate system was established on the T2 digital model. The three coordinate planes included the occlusal plane, the mid‐sagittal plane, and the coronal plane. The angles of upper first molar, canine and central incisor were measured on digital dental model using Andrews’ method [3] (Figure 4B,C). To minimize the error from landmark identification, single‐tooth superimposition was employed to transfer the landmark identified on the T0 model to the T1 and the T2 models (Figure 4D,E).

Figure 4Schematic illustration of three‐dimensional digital model superimposition and measurement definitions. (A) Superimposition of maxillary digital models from different time points. (B) Tip of the maxillary first molar. (C) Torque of the maxillary first molar. (D) Landmark a on the pre‐treatment molar. (E) Transfer of point a to the same molar on a subsequent model using regional superimposition.

**Figure:**
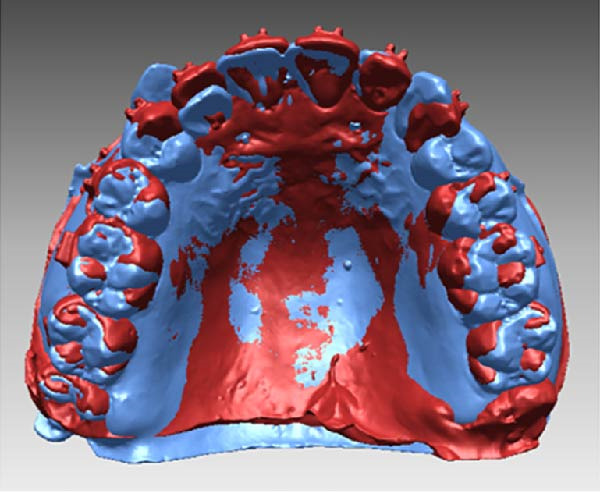
(A)

**Figure:**
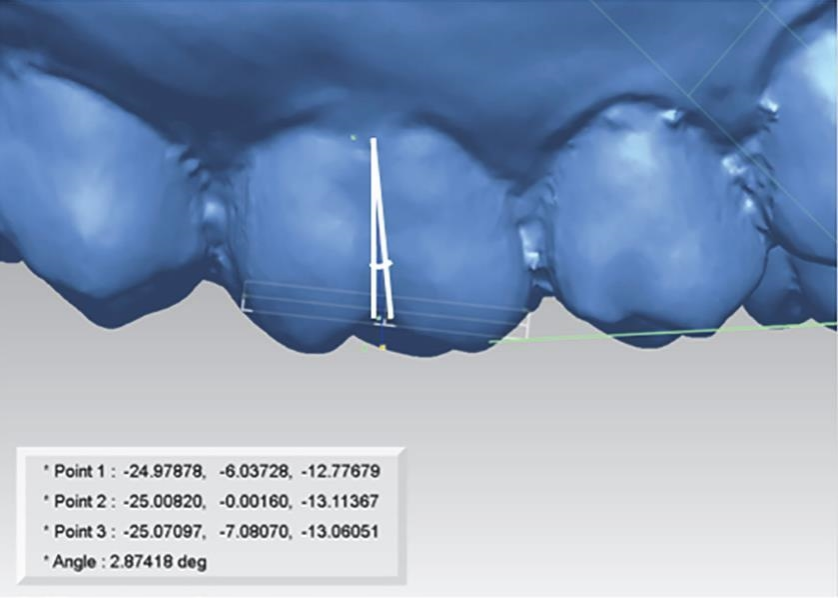
(B)

**Figure:**
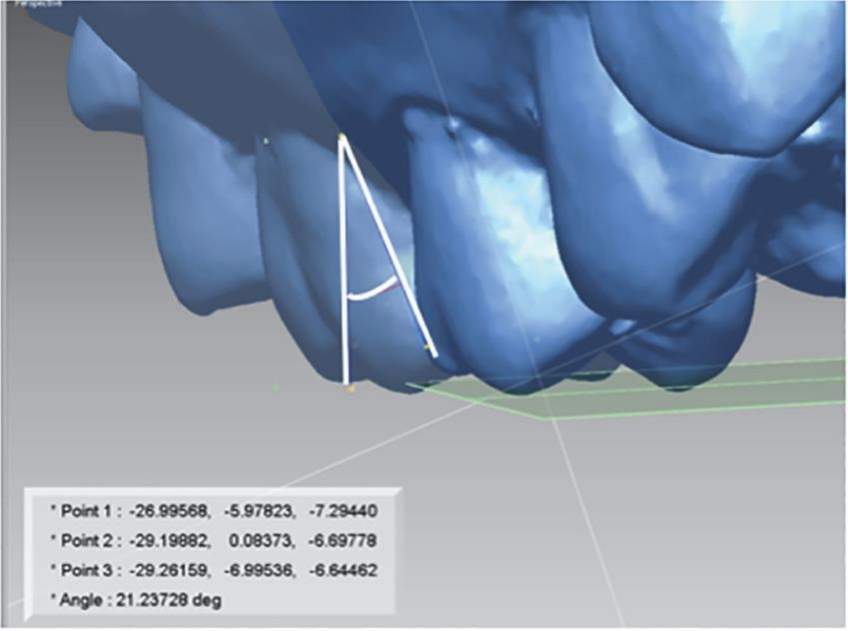
(C)

**Figure:**
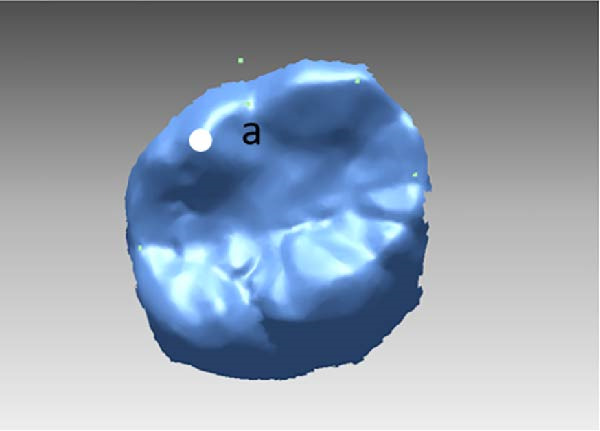
(D)

**Figure:**
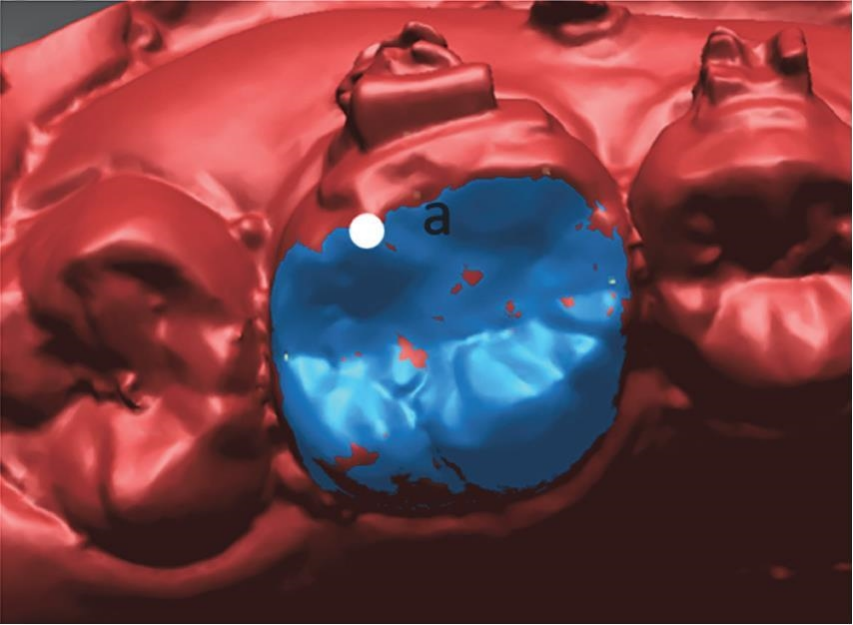
(E)

### 2.5. Sample Size Calculation

The sample size calculation was conducted with SPSS 16.0 (IBM Corp., Armonk, NY, USA), based on the known variability of the mesial displacement of upper molars measured by Xu et al. [10]. The power of the study was set at 0.80 with an alpha error probability of 0.05 to detect a mean difference of a population mean of 1.75 mm (population SD = 2.5 mm) in molar displacement [11]. The analysis indicated that a sample size of 32 participants per each group was sufficient.

### 2.6. Interim Analyses and Stopping Guidelines

No interim analyses were performed, and no stopping guidelines were implemented.

### 2.7. Randomization (Random Number Generation, Allocation Concealment, Implementation)

Patients were stratified by age, sex, and molar anchorage strength requirement. Subsequently, they were allocated into the PASS or the MBT group randomly at a 1:1 ratio using a minimization method according to the age, the sex and the molar anchorage strength [11, 18] to ensure the two groups were balanced with respect to these factors.

### 2.8. Blinding

Due to the visual similarity between PASS and MBT, patients were blind to the intervention. However, blinding of orthodontist was not feasible due to the distinct differences in appliance design and treatment procedures between the two systems. To ensure objectivity, the investigators responsible for performing the measurements and analyzing the data were blinded to group allocation.

### 2.9. Statistical Analyses (Primary and Secondary Outcomes, Subgroup Analyses)

All 192 study models were measured by three orthodontic residents. Statistical analyses were performed using SPSS 16.0 (SPSS, Chicago, IL, USA), with the significance level at *p*  < 0.05.

The Intraclass Correlation Coefficient (ICC) was calculated to assess inter‐rater consistency. Each model was measured three times by the three residents, and the mean of these measurements was used for the final analysis. The independent *T*‐test was conducted to evaluate differences between the PASS group and the MBT group. With the exception of inter‐canine width and the inter‐molar widths, all other variables were measured bilaterally. Comparisons between the right and the left sides revealed no statistically significant difference was found; thus, the bilateral measurements were combined into one measurement.

## 3. Results

### 3.1. Participant Flow

**Figure 5:**
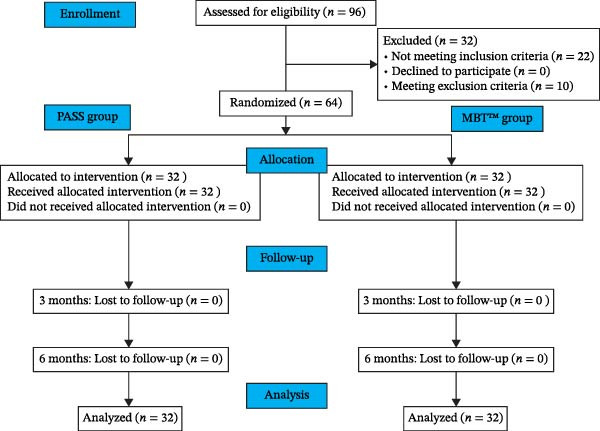
Consolidated Standards of Reporting Trials flow diagram.

A total of 64 patients were recruited and randomly allocated to the PASS group or the MBT group. No participants were lost to follow‐up during the first 3‐ and 6‐month observation periods (Figure 5).

### 3.2. Baseline Data

Stratified randomization using minimization was employed to balance variables including age, sex, and anchorage requirement (moderate or maximum). Chi‐square test analysis confirmed that the baseline characteristics was comparable between the two groups (Chi‐square = 0.07087, df = 5, *p*  > 0.99) (Table 2).

**Table 2:** Baseline characteristics of all patients.

Baseline variable	PASS	MBT	Total
Male	10	11	21
Female	22	21	43
Adolescent	22	22	44
Adult	10	10	20
Maximum anchorage	21	21	42
Moderate anchorage	11	11	22
Total	32	32	64

### 3.3. Number Analyzed for Each Outcome, Estimation, and Precision

Prior to treatments, 12 patients in the MBT group were assessed as requiring maximum anchorage for the space closure phase. In the PASS group, no auxiliary devices were utilized to enhance molar anchorage. Preliminary analyses confirmed no significant difference between the two groups the distribution of sex, age groups, and anchorage requirements.

Twenty sets of digital casts among samples were randomly selected to assess the repeatability of the 3D measurements. Superimposition and measurements were repeated after a 1‐month interval. The ICC indicated a high level of linear measurements (0.985) and angular measurements (0.886).

Significant differences were observed between the PASS and the MBT groups. The most notable difference concerned the tip of the maxillary first molar. Molars in the PASS group exhibited continuous distal tipping: −0.88° from T0 to T1, −1.79° from T1 to T2, resulting in a total of −2.67° from T0 to T2. In contrast, the molars in the MBT group initially displayed mesial tipping of the tooth crown and then slight distal tipping: 2.38° from T0 to T1, −0.43° from T1‐T2, with a total of 1.95° from T0 to T2. These differences were statistically significant. Additionally, the anterio‐posterior and vertical changes of the first molars also showed some statistical difference (Table 3).

**Table 3:** The 3D movement of the maxillary first molars, canine and central incisor in PASS and MBT groups (*n* = 64).

Measurements	Group	T0‐T1				T1‐T2				T0‐T2			
		Mean	SD	*T*	*p*	Mean	SD	*T*	*p*	Mean	SD	*T*	*p*
6–6 width (mm)	PASS	−0.61	0.89	0.123	0.902	−0.51	1.092	−2.401	0.019 ^∗^	−1.12	1.49	−1.657	0.102
	MBT	−0.65	1.07			0.11	0.96			−0.53	1.35		
6 tip (°)	PASS	−0.88	3.04	−6.492	~0.001 ^∗∗^	−1.79	3.41	−2.77	0.006 ^∗∗^	−2.67	4.59	−6.717	~0.001 ^∗∗^
	MBT	2.38	2.62			−0.43	1.96			1.95	3.04		
6 tor (°)	PASS	−1.85	6.01	−0.629	0.530	−0.5	5.84	−1.381	0.170	−2.36	5.86	−1.864	0.065
	MBT	−1.28	4.19			0.82	4.99			−0.45	5.69		
6 MCH (mm)	PASS	0.46	0.52	6.771	~0.001 ^∗∗^	0.03	0.61	1.204	0.231	0.5	0.73	6.096	~0.001 ^∗∗^
	MBT	−0.11	0.43			−0.08	0.42			−0.19	0.52		
6 DCH (mm)	PASS	0.32	0.51	2.718	0.007 ^∗∗^	−0.14	0.55	−0.034	0.973	0.18	0.67	2.129	0.035 ^∗^
	MBT	0.09	0.47			−0.14	0.38			−0.05	0.56		
6 AP (mm)	PASS	0.22	0.75	3.422	0.001 ^∗∗^	−0.24	0.96	2.063	0.041 ^∗^	−0.03	1.02	4.283	~0.001 ^∗∗^
	MBT	0.61	0.54			0.06	0.64			0.67	0.79		
3–3 width (mm)	PASS	1.04	1.53	−2.843	0.006 ^∗∗^	0.28	1.29	0.034	0.973	1.31	2.00	−2.268	0.027 ^∗^
	MBT	2.10	1.47			0.27	1.22			2.37	1.72		
3 tip (°)	PASS	−4.69	7.63	0.812	0.418	0.38	3.45	−0.233	0.816	−4.31	9.68	0.586	0.559
	MBT	−5.74	6.92			0.51	2.89			−5.22	7.86		
3 tor (°)	PASS	−1.56	7.55	−0.557	0.579	−2.08	4.08	−1.344	0.181	−3.64	8.1	−1.146	0.254
	MBT	−0.86	6.71			−1.17	3.54			−2.03	7.79		
3 H (mm)	PASS	0.32	1.44	−1.467	0.145	0.41	0.68	0.178	0.859	0.73	1.68	−1.162	0.247
	MBT	0.65	1.12			0.39	0.69			1.04	1.36		
3 AP (mm)	PASS	−2.38	1.5	−0.268	0.789	−1.19	1.43	−1.715	0.089	−3.57	2.08	−1.273	0.205
	MBT	−2.3	1.9			−0.8	1.14			−3.09	2.12		
1 tor (°)	PASS	−3.48	5.78	−1.098	0.274	−3.37	4.78	−1.375	0.172	−6.85	7.38	−1.683	0.095
	MBT	−2.41	5.24			−2.39	3.12			−4.8	6.37		
1 H (mm)	PASS	0.61	1.08	0.448	0.655	0.38	0.59	−1.01	0.314	0.99	1.17	−0.289	0.773
	MBT	0.53	1			0.53	1.04			1.06	1.47		
1 AP (mm)	PASS	−1.06	1.31	−1.388	0.168	−0.98	1.27	−0.238	0.813	−2.03	1.81	−1.112	0.268
	MBT	−0.75	1.21			−0.92	1.26			−1.67	1.88		

*Note:* “‐”: distal tipping, lingual crown torque, intrusion, distalization and reduced width.

^∗^ *p* < 0.05.

^∗∗^ *p* < 0.01.

The 3D movements of the maxillary canines and central incisors were similar bewteen the PASS and the MBT groups, with the exception of the inter‐canine width. The inter‐canine width increased by 1.31 mm in the PASS group compared to 2.37 mm in the MBT group, and it was statistically significant (Table 3).

The 3D movements of the maxillary canine and the central incisor were comparable between the adults and adolescents, both in the overall sample and within the PASS and MBT subgroups (Tables 4–6). However, adolescent patients included in the study exhibited greater mesial movement of the maxillary first molars (0.48 mm from T0 to T1; 0.49 mm total from T0 to T2) compared to adults, who showed 0.27 mm of mesial movement from T0 to T1 and 0.04 mm of distal movement from T0 to T2. The growth potential in adolescents was also associated with more mesial tipping of the maxillary first molars from T0 to T1 (Table 4). Notably, adolescent patients treated with the PASS technique exhibited similar tipping and torque from T0 to T1 (Table 5). In contrast, adolescents within MBT group demonstrated significantly greater tipping and mesial movement of maxillary molars than adults during the initial stage (Table 6).

**Table 4:** The 3D movement of the maxillary first molars, canine and central incisor in PASS and MBT group (adolescent, *n* = 44; adult, *n* = 20).

Measurements	Age	T0‐T1				T0‐T2			
		Mean	SD	*T*	*p*	Mean	SD	*T*	*p*
6–6 width (mm)	Adolescent	−0.53	1.08	1.271	0.208	−0.70	1.57	1.005	0.319
	Adult	−0.86	0.65			−1.09	1.08		
6 tip (°)	Adolescent	1.08	3.52	1.718	0.088	−0.05	4.57	1.160	0.248
	Adult	0.02	2.52			−1.05	4.38		
6 tor (°)	Adolescent	−0.77	4.44	2.645	0.009 ^∗∗^	−0.99	5.53	1.197	0.233
	Adult	−3.32	6.19			−2.32	6.41		
6 MCH (mm)	Adolescent	0.20	0.57	0.551	0.582	0.19	0.73	0.821	0.413
	Adult	0.14	0.52			0.08	0.69		
6 DCH (mm)	Adolescent	0.25	0.53	1.504	0.135	0.12	0.67	1.457	0.148
	Adult	0.11	0.43			−0.06	0.51		
6 AP (mm)	Adolescent	0.48	0.65	−1.661	0.099	0.49	0.90	−2.916	0.004 ^∗∗^
	Adult	0.27	0.72			−0.04	1.05		
3–3 width (mm)	Adolescent	1.56	1.49	−0.063	0.950	1.70	1.67	−0.884	0.380
	Adult	1.59	1.81			2.16	2.40		
3 tip (°)	Adolescent	−4.64	7.83	1.327	0.187	−3.76	9.21	1.933	0.055
	Adult	−6.48	5.74			−6.97	7.45		
3 tor (°)	Adolescent	−1.85	7.33	−1.505	0.135	−3.54	7.53	−1.480	0.141
	Adult	0.19	6.52			−1.30	8.72		
3 H (mm)	Adolescent	0.55	1.31	0.837	0.404	0.98	1.47	1.110	0.269
	Adult	0.34	1.28			0.66	1.65		
3 AP (mm)	Adolescent	−2.45	1.79	−1.139	0.257	−3.39	2.01	−0.436	0.664
	Adult	−2.08	1.50			−3.21	2.33		
1tor (°)	Adolescent	−2.61	5.11	1.029	0.305	−5.50	6.78	0.778	0.438
	Adult	−3.69	6.34			−6.54	7.32		
1 H (mm)	Adolescent	0.54	0.94	−0.403	0.688	1.00	1.33	−0.293	0.770
	Adult	0.62	1.24			1.08	1.35		
1 AP (mm)	Adolescent	−0.85	1.23	0.692	0.490	−1.85	1.88	−0.024	0.981
	Adult	−1.02	1.35			−1.84	1.79		

^∗∗^ *p* < 0.01.

**Table 5:** The 3D movement of the maxillary first molars, canine and central incisor in PASS group (adolescent, *n* = 22; adult, *n* = 10).

Measurements	Age	T0‐T1				T0‐T2			
		Mean	SD	*T*	*p*	Mean	SD	*T*	*p*
6–6 width (mm)	Adolescent	−0.60	1.02	0.123	0.903	−1.17	1.63	−0.300	0.767
	Adult	−0.64	0.54			−1.00	1.15		
6 tip (°)	Adolescent	−0.57	3.24	1.208	0.232	−2.28	4.63	1.004	0.319
	Adult	−1.56	2.51			−3.53	4.50		
6 tor (°)	Adolescent	−0.97	5.32	1.776	0.081	−2.33	5.91	0.06	0.952
	Adult	−3.80	7.07			−2.42	5.89		
6 MCH (mm)	Adolescent	0.50	0.54	0.776	0.441	0.54	0.75	0.676	0.501
	Adult	0.39	0.48			0.40	0.70		
6 DCH (mm)	Adolescent	0.37	0.54	1.223	0.226	0.25	0.72	1.228	0.224
	Adult	0.21	0.40			0.03	0.51		
6 AP (mm)	Adolescent	0.25	0.67	−0.584	0.561	0.17	0.91	−2.369	0.021 ^∗^
	Adult	0.13	0.91			−0.46	1.14		
3–3 width (mm)	Adolescent	1.04	1.57	0.004	0.996	1.36	1.97	0.206	0.838
	Adult	1.03	1.52			1.20	2.16		
3 tip (°)	Adolescent	−4.09	8.41	0.939	0.352	−3.35	10.40	1.174	0.245
	Adult	−6.02	5.48			−6.41	7.71		
3 tor (°)	Adolescent	−1.78	8.38	−0.34	0.735	−4.04	8.58	−0.576	0.567
	Adult	−1.09	5.46			−2.77	7.08		
3 H (mm)	Adolescent	0.45	1.55	1.091	0.279	0.95	1.70	1.626	0.109
	Adult	0.03	1.16			0.23	1.55		
3 AP (mm)	Adolescent	−2.54	1.49	−1.288	0.202	−3.82	2.04	−1.434	0.157
	Adult	−2.02	1.49			−3.02	2.12		
1 tor (°)	Adolescent	−2.54	6.05	1.984	0.052	−6.40	8.32	0.731	0.468
	Adult	−5.56	4.62			−7.86	4.74		
1 H (mm)	Adolescent	0.57	1.04	−0.453	0.652	0.93	1.19	−0.588	0.559
	Adult	0.70	1.18			1.12	1.15		
1 AP (mm)	Adolescent	−0.94	1.44	1.069	0.289	−2.06	1.97	−0.214	0.831
	Adult	−1.31	0.95			−1.96	1.43		

^∗^ *p* < 0.05.

**Table 6:** The 3D movement of the maxillary first molars, canine and central incisor in MBT group (Adolescent, *n* = 22; Adult, *n* = 10).

Measurements	Age	T0‐T1				T0‐T2			
		Mean	SD	*T*	*p*	Mean	SD	*T*	*p*
6–6 width (mm)	Adolescent	−0.45	1.16	1.561	0.129	−0.23	1.39	1.928	0.063
	Adult	−1.07	0.71			−1.19	1.05		
6 tip (°)	Adolescent	2.74	3.00	1.629	0.108	2.18	3.26	0.919	0.361
	Adult	1.60	1.21			1.43	2.48		
6 tor (°)	Adolescent	−0.57	3.41	2.054	0.044 ^∗^	0.35	4.83	1.695	0.095
	Adult	−2.83	5.32			−2.21	7.05		
6 MCH (mm)	Adolescent	−0.11	0.43	0.071	0.943	−0.16	0.53	0.649	0.519
	Adult	−0.11	0.45			−0.25	0.51		
6 DCH (mm)	Adolescent	0.13	0.48	0.941	0.350	−0.01	0.59	0.828	0.411
	Adult	0.01	0.44			−0.14	0.50		
6 AP (mm)	Adolescent	0.71	0.55	−2.192	0.032 ^∗^	0.80	0.77	−2.03	0.047 ^∗^
	Adult	0.40	0.44			0.38	0.77		
3–3 width (mm)	Adolescent	2.08	1.23	−0.1	0.921	2.03	1.27	−1.692	0.101
	Adult	2.14	1.98			3.11	2.35		
3 tip (°)	Adolescent	−5.19	7.26	0.931	0.356	−4.17	7.95	1.604	0.114
	Adult	−6.93	6.09			−7.53	7.33		
3 tor (°)	Adolescent	−1.91	6.20	−1.9	0.062	−3.03	6.38	−1.543	0.128
	Adult	1.46	7.36			0.17	10.07		
3 H (mm)	Adolescent	0.65	1.03	−0.029	0.977	1.02	1.21	−0.213	0.832
	Adult	0.66	1.34			1.09	1.68		
3 AP (mm)	Adolescent	−2.37	2.06	−0.429	0.669	−2.95	1.90	0.779	0.439
	Adult	−2.14	1.53			−3.40	2.57		
1 tor (°)	Adolescent	−2.68	4.03	−0.603	0.548	−4.61	4.72	0.349	0.728
	Adult	−1.82	7.32			−5.22	9.16		
1 H (mm)	Adolescent	0.52	0.83	−0.101	0.920	1.07	1.46	0.096	0.924
	Adult	0.55	1.34			1.03	1.55		
1 AP (mm)	Adolescent	−0.76	0.98	−0.133	0.895	−1.64	1.78	0.173	0.863
	Adult	−0.72	1.63			−1.73	2.13		

^∗^ *p* < 0.05.

### 3.4. Harms

No adverse events were observed in any patient in either group.

## 4. Discussion

### 4.1. Main Findings in the Context of the Existing Evidence and Interpretation

The primary finding of this trial is that the PASS technique significantly enhances molar anchorage preservation during the initial alignment and leveling stage compared to MBT. Specifically, early molar mesialization into the extraction sites was significantly minimized in the PASS group, a benefit consistently observed in both adult and adolescent patients. These results substantiate the biomechanical rationale of the PASS design regarding the control of physiological anchorage loss [19], and confirm its efficacy in preserving anchorage during the alignment phase, thereby maximizing the available space for subsequent anterior retraction.

Literature regarding anchorage loss associated with straight‐wire techniques during the alignment and leveling stage is limited. In the present study, maxillary first molars in the MBT group exhibited 1.95° ± 3.04° of mesial tipping and 0.67 ± 0.79 mm of mesial movement. In comparison, Ganzer et al. [20] reported mesial movement of the maxillary first molars of 1.2 ± 0.1 mm in a miniscrew group and 1.4 ± 0.1 mm in a control group during alignment and leveling. These values are slightly higher than the mean anchorage loss observed in our study, a discrepancy potentially attributable to the implementation of additional anchorage‐reinforcement devices (e.g., Nance appliances, headgear) in our MBT group. Consistent with the protocol described by Ganzer et al. [20], miniscrews in our study were utilized during the space closure phase rather than the alignment and leveling stage.

PASS is a novel technique in orthodontics; however, evidence regarding its efficacy in controlling molar anchorage during the alignment and leveling stage is currently lacking. This study presents a novel comparison of anchorage preservation during the early alignment and leveling stage between the PASS and MBT systems. In contrast to the mesial movement observed in the MBT group, the maxillary first molars in the PASS group exhibited 2.67° of distal tipping and 0.03 mm of distal displacement at the mesiobuccal cusp within the first 6 months, demonstrating effective preservation of molar anchorage. These results offer insight into the mechanism underlying the superior anchorage control of the PASS technique, as observed in Chen’s randomized controlled trial [11], even without auxiliary anchorage reinforcement. Consequently, these findings raise an important question: why does the PASS technique exhibit superior early molar anchorage preservation compared to the MBT system?

We investigated the factors contributing to the observed differences in anchorage control between the PASS and the MBT systems. The buccal tube prescription in conventional straight‐wire technique typically incorporates a mesial inclination, which may predispose the molar to mesial tipping and lead to early anchorage loss [12]. Furthermore, the use of lacebacks for canine retraction and the limitation of incisor proclination remain controversial regarding their potential to induce molar anchorage loss [21]. The PASS technique employs a differential moment derived from Burstone’s six classes of two‐tooth force systems [22], rather than relying on laceback mechanics for canine movement, thereby avoiding potential anchorage reduction. Designed in accordance with the physiological characteristics of the jaws, PASS leverages the natural anchorage potential of the molars by applying a differential moment between the canine and the molar; this facilitates distal canine migration while simultaneously preventing molar mesialization. In the PASS protocol, second premolars are left unbonded during the initial alignment and leveling stage, effectively increasing the inter‐bracket span and moment arm. Upon insertion of the initial archwire into the tip‐back tube, this configuration facilitates the generation of a protective distal moment on the molar from the onset of treatment. This favorable molar‐canine moment is absent in the MBT system due to differences in appliance design and treatment protocols. Consequently, this design reinforces distal crown tipping, even when using light NiTi wires and without the need for manual wire bending. Although the moment may not be of sufficient magnitude to actively tip the molars distally, it maintains their original angulation and counteracts physiological anchorage loss associated with growth and development. The omission of premolar bonding at this stage facilitates the generation of a favorable molar‐canine moment [23]. When the NiTi archwire is engaged from the anterior brackets into the −25° tip‐back tube, it does not undergo deformation. This −25° tip‐back tube produces a biomechanical effect analogous to a V‐bend positioned close to the first molar, generating a clockwise moment on both the first molar and the canine. Therefore, the physiological curve of Spee is maintained.

We further identified the specific timing of anchorage loss. By evaluating the maxillary molar movement at specific intervals, we observed that molars tipped distally by ~1° and moved mesially by 0.2 mm in the PASS group during the first 3 months. This differed significantly from the MBT group, which exhibited 2.4° of mesial tipping and 0.6 mm of mesialization. The MBT group experienced rapid anchorage loss during this period, accounting for the majority of the total loss observed over the first 6 months. This may be attributed to the mechanical properties of the initial archwires; the thin NiTi wires (0.012–0.014 inch) utilized at this stage may lack sufficient rigidity to completely counteract molar mesialization tendencies. However, the −25° molar tube in the PASS system effectively prevented mesial tipping of the maxillary molars. After 3 months of treatment, stiffer NiTi archwires (0.016–0.018 inch) were introduced. Therefore, compared with the MBT technique, the PASS effectively minimized anchorage loss throughout the entire alignment and leveling stage. Given that the anchorage loss has been reported to be associated with age and growth [24], we evaluated maxillary first molars movement in both adolescent and adult patients. In the PASS group, the mesial displacement of the mesiobuccal cusp during the first 3 months was similar between adolescents (0.25 ± 0.67 mm) and adults (0.13 ± 0.91 mm). In contrast, adolescents in the MBT group exhibited greater mesial movement (0.71 ± 0.55 mm) compared to adults (0.40 ± 0.44 mm). These results indicate that the PASS technique can effectively resist unfavorable anchorage loss associated with growth during the first 3 months of alignment and leveling. While skeletal anchorage devices, such as miniscrews, provide maximum to absolute anchorage [25], the failure rate of these devices is higher in adolescents than in adults [25], a finding associated with active bone turnover and remodeling [26]. Thus, by effectively reducing anchorage loss in adolescents, the PASS technique avoids the potential pain and discomfort caused by miniscrew failure in patients requiring reinforced anchorage.

With respect to canine changes between the two groups, a statistically significant difference was observed in inter‐canine width. This difference is primarily attributable to variations in treatment protocols. The MBT system involves bonding the second premolars at the onset of treatment, whereas the PASS technique does not.

Typically, the mesial displacement of the first molar is sufficient to evaluate anchorage loss. However, the situation differs when comparing anchorage control during alignment and leveling, as the extraction spaces have not yet been closed in most cases. In this context, the magnitude of central incisor movement is a critical factor. Since no statistically significant difference was observed in the movement of the central incisors between the two groups, it is valid to evaluate anchorage control solely by comparing the positional changes of the first molars in the MBT and PASS groups.

### 4.2. Limitations

The present study was conducted at a single center. Although the inclusion of an untreated control group would be ideal to differentiate treatment effects from normal growth changes, this was not feasible because physiological drift of the anchorage molars would inevitably occur during the observation period.

## 5. Conclusions

In the MBT group, upper first molars exhibited significant forward tipping during the alignment and leveling, particularly within the first 3 months, indicating that anchorage loss primarily occurs at the beginning of the treatment. Conversely, in the PASS group, upper first molars showed continuous distal tipping during alignment and leveling, resulting in effective molar anchorage preservation. Consequently, the null hypothesis that the PASS and MBT systems possess similar capacity of anchorage capacities during the initial stage of treatment was rejected. The PASS technique is superior in reducing molar mesialization and mesial tipping during alignment and leveling, thereby providing enhanced anchorage preservation.

## Author Contributions

Conceptualization, Tianmin Xu; methodology, Gui Chen; formal analysis, Bing Han and Tingting Feng; investigation, Kaiyuan Xu, Zimeng Zhuang and Fei Teng; resources, Hong Su and Gui Chen; data curation, Hong Su, Kaiyuan Xu and Fei Teng; writing—original draft preparation, Hong Su; writing—review and editing, Bing Han and Zimeng Zhuang; supervision, Gui Chen; project administration, Tianmin Xu and Gui Chen. All authors have read and agreed to the published version of the manuscript.

## Funding

This research was funded by the National Natural Science Foundation of China (grant number 82571131, 82071172), the Program for New Clinical Techniques and Therapies of Peking University School and Hospital of Stomatology [grant number PKUSSNCT‐22B01], Beijing Natural Science Foundation (grant number L242134, L252199).

## Disclosure

A preprint has previously been published [27, 28].

## Ethics Statement

The study was conducted according to the guidelines of the Declaration of Helsinki, and approved by the Biomedical Ethics Committee of the School and Hospital of Stomatology, Peking University (Beijing, China) and registered at the Chinese Clinical Trial Registry (Chictr.org.cn) (protocol code ChiCTR‐TRC‐13003260, 15/06/2013).

## Consent

Informed consent was obtained from all subjects involved in the study. Written informed consent has been obtained from the patient(s) to publish this paper.

## Conflicts of Interest

No potential conflict of interest relevant to this article was reported. The fundings provided did not affect the neutrality, objectivity or assessment of this article.

## Data Availability

The data that support the findings of this study are available from the corresponding author upon reasonable request.
